# ABCB5: A Key Regulator Linking Stem Cell Plasticity, Tumor Microenvironment, and Therapy Resistance in Cutaneous Melanoma

**DOI:** 10.3390/cancers18030424

**Published:** 2026-01-28

**Authors:** Andreea Cătălina Tinca, Adrian Horațiu Sabău, Andreea Raluca Cozac-Szoke, Diana Maria Chiorean, Bianca Andreea Lazar, Raluca-Diana Hagău, Iuliu Gabriel Cocuz, Raluca Niculescu, Irina Bianca Kosovski, Sofia Teodora Muntean, Sabin Gligore Turdean, Ovidiu Simion Cotoi

**Affiliations:** 1 Pathophysiology Department, George Emil Palade University of Medicine, Pharmacy, Science and Technology, 540142 Târgu Mureș, Romania; andreea-catalina.tinca@umfst.ro (A.C.T.); andreea.szoke@umfst.ro (A.R.C.-S.); diana.chiorean@umfst.ro (D.M.C.); iuliu.cocuz@umfst.ro (I.G.C.); raluca.niculescu@umfst.ro (R.N.); bianca.kosovski@umfst.ro (I.B.K.); sofia.harsan@umfst.ro (S.T.M.); ovidiu.cotoi@umfst.ro (O.S.C.); 2 Pathology Department, Clinical County Hospital Mureș, 540136 Târgu Mureș, Romania; ohii.bianca@yahoo.com (B.A.L.); ralucahagau1995@gmail.com (R.-D.H.); sabin.turdean@umfst.ro (S.G.T.)

**Keywords:** melanoma, ABCB5, tumor microenvironment, therapy resistance

## Abstract

Cutaneous melanoma is one of the most aggressive skin cancers. Over the years, multiple studies have focused on identifying novel treatment strategies, with increasing attention directed toward immune-modulating mechanisms within the tumor microenvironment. Among these, ATP-binding cassette transporters and stem-associated pathways have been shown to influence drug response and immune escape. ABCB5 is a gene with multiple isoforms that significantly influences the immune response. In melanoma, the ABCB5α isoform is predominantly expressed, particularly in tumor stem-like cells where it promotes chemoresistance through active drug efflux. ABCB5 has also been linked to the regulation of PI3K/Akt, BCL-2, and miR-145-associated pathways. Moreover, ABCB5-positive cells contribute to the formation of an immunosuppressive microenvironment by secreting cytokines (IL-6, IL-8, TGF-β) and expressing immune checkpoint ligands, such as PD-L1, thereby favoring tumor progression and a poor prognosis. This review integrates current data on the molecular and microenvironmental mechanisms underlying melanoma progression and therapy resistance, and positions ABCB5 within the broader landscape of melanoma resistance mechanisms, emphasizing both its potential and its current limitations as a biomarker and therapeutic target.

## Simple Summary

Cutaneous melanoma is one of the most aggressive forms of skin cancer and remains difficult to treat due to its ability to adapt and develop resistance to therapy. Recent research suggests that a small subset of tumor cells with stem-like properties play an important role in disease progression and treatment failure. One molecule of growing interest is ABCB5, a membrane transporter associated with drug resistance, immune evasion, and tumor survival. This review summarizes current knowledge on how ABCB5 contributes to melanoma progression by influencing tumor cell plasticity and the surrounding tumor microenvironment. We discuss the biological mechanisms linked to ABCB5 expression, its potential value as a prognostic biomarker, and its relevance for future therapeutic strategies. A better understanding of ABCB5 may help guide the development of more effective and personalized treatment approaches for patients with melanoma.

## 1. Introduction

**Figure 1:**
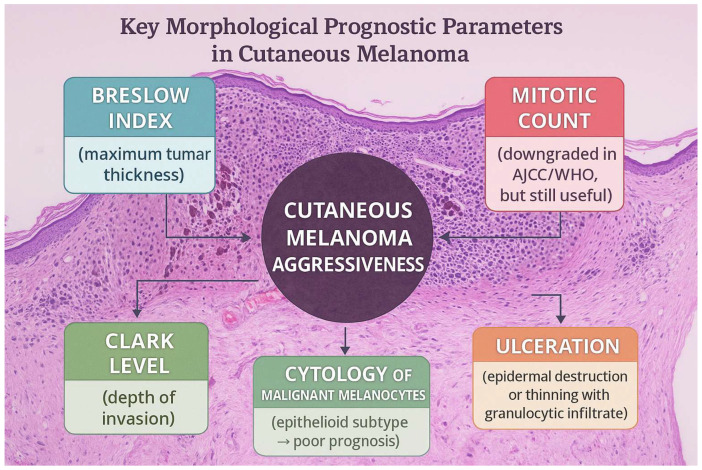
Prognostic factors in cutaneous melanoma.

Cutaneous melanoma is one of the most aggressive cancers, whose aggressiveness depends on important morphological parameters with prognostic value. The American Joint Committee on Cancer (AJCC) and World Health Organization (WHO) guidelines identify high Breslow index, ulceration, high mitotic count, and epithelioid cytology as features associated with poor prognosis. Of great prognostic importance is the presence of satellites or microsatellites (groups of tumor cells located within 2 cm of the primary tumor, first identified on gross examination and the second detected under the microscope) (Figure 1). Identifying these aspects alters the tumor’s pTNM stage, placing the patient in the Nc category, irrespective of lymph node status. Such a change in the pTNM also occurs if the tumor nodules are detected at a distance greater than 2 cm from the primary tumor, indicating the presence of in-transit metastasis [1,2,3].

Due to the tumor’s poor prognosis and continuously increased incidence in the general population all around the Globe, various therapies have been developed in the past decades. A significant discovery was made in the early 2000s, when BRAF (B-Raf proto-oncogene, serine/threonine kinase) and its role in melanoma development were described. Thus, based on the data gathered at the time, targeted therapy was invented [4,5].

Metastatic melanoma has a very poor prognosis. This form is very resistant to chemotherapy, radiation therapy, or targeted therapy and represents a constant challenge for oncologists. Despite major advances in targeted therapy and immunotherapy, cutaneous melanoma remains characterized by significant therapeutic resistance and disease progression, largely driven by tumor heterogeneity and cellular plasticity. Recent evidence highlights the contribution of stem-like tumor cell populations and microenvironment-mediated immune suppression as central mechanisms underlying treatment failure, underscoring the need for improved biomarkers and novel therapeutic targets [1,2,5].

This review aims to integrate molecular, microenvironmental, and clinical evidence to critically assess the role of ABCB5 in melanoma progression and therapy resistance, with particular emphasis on unresolved controversies and translational challenges. Important pathophysiological, molecular, and therapeutic data on melanoma are presented to provide a more straightforward overview and understanding. Emphasis is placed on the biological mechanisms underlying ABCB5 expression, its potential as a biomarker, the existing controversies in the literature, and its possible therapeutic implications.

The inclusion of general melanoma biology and microenvironmental features is intended to provide a conceptual framework and place the functional role of ABCB5 in melanoma progression and therapeutic resistance in context.

## 2. Search Strategy and Study Selection

This review was based on a structured literature search conducted in PubMed, Scopus, and Web of Science. The research employed Boolean operators to combine keywords such as ABCB5, melanoma, melanoma stem cells, immunotherapy, therapy resistance, and tumor microenvironment. The search covered articles published between 2000 and 2025, and we primarily included English-language articles, with selected non-English references included when essential to the topic. Peer-reviewed original research articles, translational studies, and clinical studies were considered, as well as relevant review articles when necessary to provide contextual background. Studies unrelated to melanoma, molecular pathways, microenvironment, treatment, and ABCB5 were excluded. Article selection was performed by two authors based on title and abstract screening, followed by full-text assessment of relevant publications. Discrepancies were resolved by consensus, and we included a total of 87 articles. Given the narrative nature of the review, no formal quality assessment or PRISMA flow diagram was applied.

Limitations: This review is narrative in nature and is therefore subject to selection bias. Heterogeneity among experimental models and clinical cohorts limited direct comparison across studies.

## 3. Tumor Development

**Figure 2:**
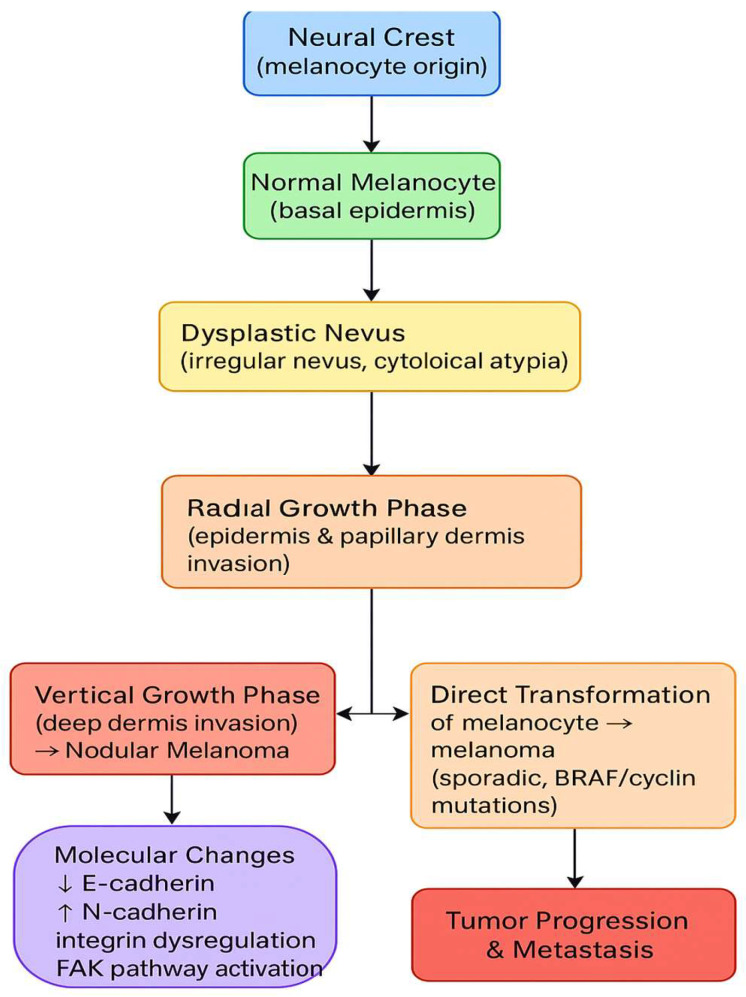
Stages in the development of melanoma.

Melanoma development is characterized by marked cellular plasticity and molecular heterogeneity, resulting from the dysregulation of normal melanocyte differentiation and cell–cell interactions. These alterations enable tumor cells to switch phenotypic states, invade surrounding tissues, and adapt to therapeutic pressure. Such plasticity represents a key biological foundation for the emergence of stem-like tumor cell populations and the development of resistance to both targeted therapy and immunotherapy (Figure 2) [6,7,8,9].

## 4. Tumor Microenvironment

The tumor microenvironment (TME) plays a central role in melanoma tumorigenesis and therapeutic response. It comprises stromal components, including fibroblasts, the extracellular matrix, blood vessels, inflammatory cells, and soluble factors, that collectively support tumor growth and immune modulation. In melanoma, marked intratumoral and intertumoral heterogeneity, including the variable expression of HLA class I molecules and immune regulators, such as PD-L1, significantly influences tumor behavior and therapy resistance [10,11,12,13,14,15]. Within this context, ABCB5-positive melanoma cells have been implicated in shaping an immunosuppressive microenvironment through cytokine secretion and immune checkpoint modulation.

Microphthalmia-associated transcription factor (MITF), which governs melanocyte proliferation, differentiation, and survival, is a key transcriptional regulator in melanoma. High MITF expression is associated with proliferative melanoma states, whereas low or intermediate MITF expression is linked to invasive, drug-tolerant phenotypes [16,17,18]. MITF expression is tightly regulated by multiple transcription factors, including CREB and SOX10, which cooperatively activate the MITF promoter [19,20].

Beyond its role in melanocyte development, the MITF–PAX3 regulatory axis is a critical determinant of melanoma cell plasticity. PAX3 regulates MITF at both the transcriptional and functional levels and is essential for maintaining progenitor-like characteristics. MITF-driven transcriptional states have been linked to melanoma phenotype switching, which is closely associated with therapy resistance and tumor adaptation. Emerging evidence suggests that ABCB5-positive melanoma cells preferentially occupy low-MITF or intermediate-MITF states, which is consistent with a stem-like, drug-tolerant phenotype. In this context, ABCB5 expression may reflect or stabilize MITF-associated transcriptional programs that favor cellular plasticity, survival signaling, and resistance under therapeutic pressure [21,22].

PAX3 expression is modulated by TGF-β and interleukin-6 receptor signaling and lies downstream of the Hippo pathway. The Hippo effectors YAP and TAZ function as transcriptional cofactors for PAX3, linking mechanical cues from the extracellular matrix to MITF regulation. This axis plays an important role in melanoma cells’ adaptation to microenvironmental stress and may contribute to the maintenance of quiescent, stem-like tumor cell populations [22,23,24,25,26,27,28,29]. Given the association of PAX3-driven programs with progenitor features and resistance to apoptosis, ABCB5 expression may indicate a PAX3-permissive cellular state that contributes to tumor persistence and therapeutic resistance.

FOXD3 is another transcription factor that is involved in melanoma plasticity, acting primarily as an MITF repressor by blocking PAX3 via binding to its promoter. In melanoma, FOXD3 expression has been linked to resistance to BRAF inhibition through the suppression of MITF and the upregulation of ERBB3 signaling [30,31,32,33,34]. As FOXD3 is associated with drug-tolerant melanoma states, FOXD3-driven programs may partially overlap with ABCB5-positive phenotypes, rather than directly regulating ABCB5 expression.

SOX10 is essential for melanocyte lineage maintenance and melanoma development and directly cooperates with PAX3 to activate MITF transcription. In BRAFV600E-mutant melanoma, the ERK-mediated inhibition of SOX10 activity contributes to reduced MITF expression and phenotype switching under therapeutic pressure [35,36,37,38,39]. SOX10 also interacts with PGC1α, forming transcriptional circuits that integrate environmental signals with melanoma cell identity. SOX10-dependent transcriptional programs influence melanoma cells’ survival, immune interactions, and lineage stability within the TME. ABCB5-positive melanoma cells have been associated with immune-modulatory features, including cytokine secretion and PD-L1 upregulation, suggesting that SOX10-regulated states may intersect with ABCB5-associated immune evasion mechanisms [40,41,42,43,44].

Tumor-infiltrating lymphocytes (TILs) are key modulators of the melanoma microenvironment and have prognostic significance. Brisk lymphocytic infiltration is associated with improved outcomes, whereas reduced immune infiltration indicates impaired antitumor immunity and a worse prognosis [45,46,47]. Notably, ABCB5-positive melanoma cells have been linked to immunosuppressive signaling and reduced immune infiltration, offering further support for ABCB5’s role in shaping an adverse tumor–immune landscape.

## 5. Metastatic Melanoma

Metastatic melanoma carries a poor prognosis. Despite nodular melanoma accounting for most metastatic cases, some studies describe superficial spreading melanoma as the leading cause. Therefore, despite the Breslow index being the most important prognostic parameter for tumor morphology, thin melanomas and melanomas with radial growth also have a high metastatic potential. Among melanoma subtypes associated with a high risk of progression, we encounter lentigo maligna melanoma and acral melanoma [48,49].

From a clinical perspective, patients may present with solitary or multiple cutaneous lesions. The risk factors associated with metastatic melanoma are the male gender, the location of the tumor (head and neck tumors), lymphatic and vascular invasions, and the presence of BRAF mutation [50].

From a histological point of view, metastatic melanoma can present with a high morphological variation. Cells may present with characteristics similar to those of the primary tumor or be highly heterogeneous and pleomorphic, complicating diagnosis. In these cases, the differential diagnosis is challenging, and a variety of immunohistochemistry markers must be performed for confirmation. Melanoma is an excellent mimic of other entities, sometimes resembling poorly differentiated carcinomas and various sarcomas. Melanoma typically expresses markers such as SOX10, S100, HMB45, MelanA, MITF, or PRAME and is negative for myogenic or epithelial markers. The challenge arises when the tumor is dedifferentiated and loses its classic positivity for these markers. One of the most specific markers is SOX10, which is widely used to confirm the diagnosis and for sentinel lymph node protocols. This marker is not specific to melanoma, but it is susceptible and shows higher specificity than other markers. The use of multiple melanocytic markers together can significantly increase diagnostic accuracy. Lately, PRAME has been tested and used for primary and metastatic melanoma and is thought to have a good sensitivity to the tumor. However, this new marker alone is not the most reliable for distinguishing primary malignant melanocytic neoplasms and should be combined with other markers. For metastatic melanoma, PRAME can be expressed even in certain dedifferentiated areas, aiding significantly in revealing the tumor’s origin [51,52,53,54,55].

Dedifferentiated metastatic melanoma can present a part of the tumor that still preserves the classic aspect of melanoma, but most of the neoplasm can be far from the conventional aspect. This dedifferentiated component can resemble an undifferentiated sarcoma or pleomorphic sarcoma. When all IHC markers, including PRAME, are negative, molecular analysis is mandatory. The most common mutations are BRAF V600E or V600K, which are identified in half of melanoma cases. Other mutations that can be encountered include NRAS Q61, especially in desmoplastic melanoma or in cases without a BRAF mutation. KIT mutations in exons 11 or 13 are commonly observed, particularly in acral melanoma. NF1 can also be identified and is most commonly observed in triple-negative melanoma (BRAF-, NRAS-, and KIT-negative). The most helpful tool is NGS (Next-Generation Sequencing), which can analyze multiple mutations, fusions, or amplifications. Other techniques that can be useful are FISH (Fluorescent in situ hybridization) and mutational signatures. Mutational signatures are determined from tumor DNA, which undergoes whole-genome sequencing to identify somatic mutations consistent with UV exposure. This method is essential and innovative but also has several limitations and lacks clinical standardization; therefore, it is used primarily in research [56,57,58,59].

## 6. Therapeutic Approaches in Cutaneous Melanoma

### 6.1. Localized Disease

For early-stage melanoma (pT1, pT2), the primary treatment is surgical wide excision with negative margins. Surgery is mandatory not only as a therapeutic option but also as a diagnostic tool, as histopathological examination is the gold standard for diagnosis. The guidelines emphasize the importance of lymph node status; therefore, sentinel lymph node excision and examination are usually performed after surgery [60].

### 6.2. Advanced Disease and Targeted Therapy

The systemic melanoma treatment landscape has undergone significant changes since the beginning of the century, when BRAF was discovered. The previous therapeutic options for advanced stages mainly consisted of chemotherapy and sometimes radiation therapy. Dacarbazine played an essential role despite its numerous side effects. Platinum-based drugs like carboplatin were also used in combination with multiple other medicines, including alkylating agents or dacarbazine. Radiation therapy could also be an option, being reserved for cases with extended lymph node involvement [61].

The discovery of BRAF in 2002 led to the development of targeted therapy. Of the most common drugs used, Vemurafenib has held the spotlight for the longest. However, the resistance to this type of treatment has been reported in many cases. This is observed in nearly half of patients and is primarily attributable to intratumoral heterogeneity. Cases that do not present BRAF mutation also benefit from therapy, but with a less pronounced response [62,63,64].

### 6.3. Advanced Disease and Immunotherapy

The treatment of metastatic melanoma has also changed significantly in the last decade, with the development of targeted therapy and immunotherapy. Without modern treatment, the overall survival used to be 6 to 9 months, and less than 10% of the patients survived for 5 years. Following the development of immunotherapy, survival has increased to more than 50 months within the first 5 years, and some studies report a 6-year survival rate. The first line of treatment in patients with high LDH levels and visceral metastases is represented by anti-CTLA-4 and anti-PD-1 medication, such as ipilimumab and nivolumab. These options can significantly increase survival; in half of patients, survival is estimated at 7 years [64,65].

A possible combination is nivolumab and relatlimab, an anti-LAG-3 agent that is generally better tolerated. Patients with BRAF mutations can be treated with BRAF inhibitors and achieve rapid responses; however, in the long term, resistance is common. In such cases, immunotherapy is preferred, or various combinations of agents are used [65].

### 6.4. Cellular and Emerging Therapies

When patients do not respond to these therapies, innovative options are considered. One of these is lifileucel, a therapy that uses isolated infiltrating T lymphocytes (TIL) from the patient’s tumor. These lymphocytes are then reinfused to attack the tumor cells. The steps include the excision of the metastatic tumor, the isolation and cultivation of lymphocytes, the administration of chemotherapy to suppress the immune response, and the reinfusion of the lymphocytes [65]. Lifileucel received regulatory approval by the U.S. Food and Drug Administration (FDA) in 2024 for the treatment of unresectable or metastatic melanoma in patients who had progressed after prior anti-PD-1 therapy [66].

The key to a therapeutic success lies in the cells’ ability to recognize tumor antigens and attack tumor cells. Available studies showed increased survival over 2 years in this category of cases. Other options under investigation include toll-like receptors 9 and 7/8, stimulators of interferon genes, and agonists. These studies aim to modulate, prevent, and regulate the immune system response to the tumor [67].

Despite these advances, a substantial proportion of patients develop resistance to both targeted therapies and immunotherapy. Increasing evidence suggests that therapy resistant melanoma cell subpopulations with stem-like features may persist despite treatment and contribute to disease recurrence. In this context, ABCB5 has been implicated in drug efflux, survival signaling and immune evasion, potentially influencing response to both conventional and emerging therapeutic options. Although ABCB5 is not currently used as a clinical biomarker, its expression may help identify resistant tumor cell populations and support patient stratifications in the future [65,68].

## 7. Stem Cells in Melanoma

Melanoma stem cells (MSCs) are a distinct population of cells that can self-renew and are directly involved in disease progression. Their existence has long been a topic of interest in dermatopathology and oncology, but they have ultimately been identified using various markers. These specialized cells are considered to express CD271 (NGFR), CD133, ALDH1, Nestin, and CD44. Compared to these markers, ABCB5 is distinctive in that it combines stem-associated properties with functional drug efflux capacity and immune-modulatory effects. ABCB5 expression is heterogeneous and context-dependent, highlighting both its potential and its limitations as a standalone stemness marker. The plasticity of these stem cells is remarkable, as they can exist in a proliferative or a stem-like state. The cells are activated by factors such as hypoxia, ischemia, inflammation, oxidative stress, and chemotherapy. Thus, MSCs are not a rigid population; they are cells in a constant state of reprogramming that can adapt to the environment. Functionally, the cells exhibit high resistance to chemotherapy and immunotherapy, express efflux transporters and checkpoint molecules (e.g., PD-L1), and synthesize immunomodulatory factors that interfere with the T cell response in the microenvironment [69,70,71].

## 8. ABCB5

### 8.1. ABCB5 Biology

ABCB5 was initially identified as a human P-glycoprotein homolog in 1996, but functional studies of ABCB5 in melanoma stem cells did not occur until the mid-2000s. The gene encodes ATP-binding cassette (ABC) transporter family subtype B member 5, a protein with multiple isoforms. Pathophysiologically, ABCB5 was considered for its role as a tumor marker, specifically, a stem cell marker, and in cancer biology [68,72].

Mesenchymal cells can differentiate into multiple lineages of the connective tissue. They are essential for producing the extracellular matrix, interacting with the immune system, and synthesizing important homeostasis molecules [73].

**Figure 3:**
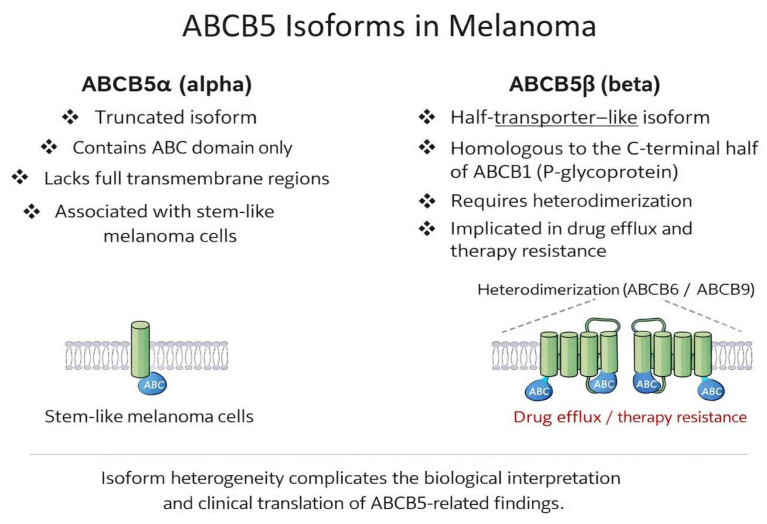
Schematic representation of ABCB5 isoforms and their proposed functional roles in melanoma.

The most-studied isoform, predominantly expressed in melanoma, is ABCB5α; other isoforms are primarily associated with processes such as glycolysis or atherosclerosis. The isoforms associated with melanoma and their roles are shown in Figure 3.

ABCB5 has been identified on MSCs, and it is expressed in tumor cells with stem-like features. ABCB5 is predominantly localized to the cell membrane, consistent with its transporter function, although cytoplasmic expression has also been reported in certain experimental settings. The biological and clinical significance of ABCB5’s subcellular localization in melanoma remains unclear and warrants further investigation [74,75]. ABCB5 appears to reduce drug accumulation within cells. Some studies report that ABCB5-expressing cells persist after treatment with temozolomide, dacarbazine, and vemurafenib, both in vitro and in vivo, despite a decrease in overall tumor burden. ABCB5-expressing cells are also more common in tumor samples from treated patients. Together, these findings suggest that ABCB5+ cells are more resistant to therapy and may contribute to disease recurrence [74].

Melanoma exhibits high intratumoral heterogeneity, and ABCB5 contributes to the formation of distinct cell subpopulations with differential proliferative and invasive capacities. ABCB5+ cells can coexist with ABCB5− cells, creating a heterogeneity that influences therapeutic response, as positive cells can persist after treatment and repopulate the tumor [73,74,76].

### 8.2. Mechanism and Roles

The pathophysiological mechanisms underlying ABCB5 modulation of treatment responses are closely linked to MSC plasticity. ABCB5+ cells exhibit well-controlled differentiation and a high renewal rate; these traits increase the recurrence and long-term survival of tumor cells. The primary molecular mechanism by which ABCB5 supports cell survival is active drug efflux. In addition, ABCB5 regulates cellular homeostasis and facilitates tumor cells’ evasion of therapy-induced cell death. This is achieved by modulating the expression of antioxidant genes and pathways, such as PI3K/Akt [77,78].

High ABCB5 expression is also associated with low apoptosis, a phenomenon that is attributable to changes in BCL-2 protein expression. Recent studies have shown that ABCB5 is regulated by miRNAs, which can alter tumor resistance phenotypes. When miR-145 is expressed at normal levels, it suppresses ABCB5. This is associated with tumor cells’ inability to extrude drugs. However, in advanced melanoma, miR-145 levels often decrease, resulting in ABCB5 overexpression and increased therapy resistance. Restoring miR-145 to normal levels could increase cells’ sensitivity to standard treatment [76,78].

**Figure 4:**
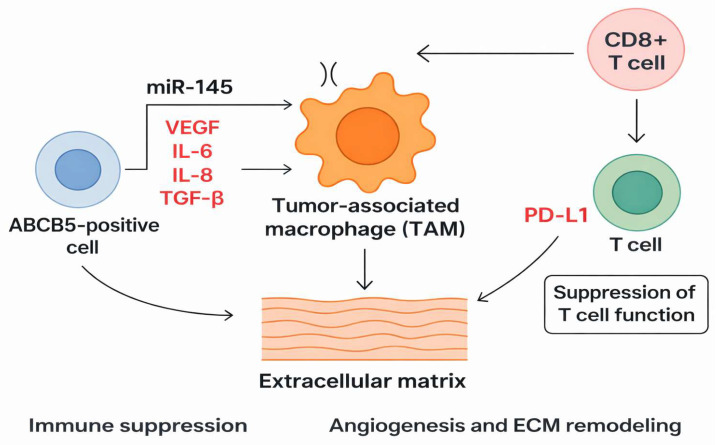
Schematic representation of the interactions between ABCB5-positive melanoma cells and the tumor microenvironment. ABCB5-positive cells promote immune suppression and angiogenesis by modulating tumor-associated macrophages (TAMs) and by remodeling the extracellular matrix, involving mediators such as VEGF, IL-6, IL-8, and TGF-β. The regulatory role of the miR-145–ABCB5 axis is also illustrated, mechanism detailed in the text. Arrows indicate the directionality of regulatory interactions rather than quantitative magnitude.

ABCB5+ cells can influence the microenvironment by synthesizing angiogenic and inflammatory factors that support tumor growth. These factors are VEGF (vascular endothelial growth factor), IL-6 (interleukin 6), IL-8 (interleukin 8), and TGF-β (tumor growth factor beta). The interaction between ABCB5+ cells, T cells, and macrophages favors immune suppression and a lack of antigen recognition. Tumor-associated macrophages (TAMs) will present an M2 phenotype due to the cytokines secreted by ABCB5+ cells. This phenotype is associated with the suppression of the adaptive immune response, the further promotion of angiogenesis, and the remodeling of the extracellular matrix. Interaction with T cells, particularly CD8+ T cells, leads to the overexpression of PD-L1 and suppression of T-cell function (Figure 4) [79].

Some studies report that ABCB5 is overexpressed in BRAF inhibitor-resistant melanoma cell lines. The expression patterns varied by drug type and treatment stage, highlighting inter-cell-line heterogeneity. However, ABCB5 knockdown did not restore vemurafenib sensitivity, suggesting that it is not a primary driver of BRAF resistance. ABCB5 expression was correlated with increased phosphorylated ERK levels (a protein in the MAPK pathway), but the inhibition of p-ERK, not ABCB5, reversed resistance. These findings, along with previous studies showing variable ABCB5 expression and ABCB5 co-regulation with other resistance genes, suggest that although it contributes to chemoresistance and tumor heterogeneity, it may not be essential for BRAF inhibitor resistance [75,79,80].

In melanoma, ABCB5 expression cooperates with other transporters, such as ABCB1 and ABCG2. This co-expression has been associated with multidrug-resistant phenotypes and metabolic reprogramming. These cooperative networks must be understood to develop combination therapies that can overcome resistance [81].

### 8.3. Clinical Significance

Several retrospective tissue-based studies have reported an association between high ABCB5 expression and aggressive disease features, including reduced overall survival, increased recurrence rates, and metastatic potential. However, most of the available evidence was derived from in vitro experiments, animal models, and retrospective analyses of human tissue samples, while prospective clinical validation and multivariate analyses remain limited. Patients with high ABCB5 expression experienced decreased overall survival, frequent recurrence, and an increased risk of distant metastases. ABCB5 may serve as a marker of chemotherapy resistance as it reflects the tumor’s ability to evade the immune response (Table 1) [82,83]. However, ABCB5 expression’s correlation with established histopathological parameters, such as Breslow thickness, ulceration status, and tumor-infiltrating lymphocytes, remains poorly characterized and lacks homogenous data, underscoring the need for standardized clinicopathological studies [76,84].

### 8.4. Limitations of the Current Evidence and Open Questions

Despite growing interest in ABCB5 as a biomarker and therapeutic target, several significant limitations and questions remain. As noted above, ABCB5 is expressed as multiple isoforms, and their relative biological roles in melanoma remain incompletely understood [74]. This structural heterogeneity complicates biological interpretation and clinical translation. From a technical perspective, variability in antibody specificity and staining protocols represents a significant challenge for the immunohistochemical interpretation of ABCB5, contributing to inconsistent experimental results [83,85,86]. In addition, although ABCB5 expression is associated with BRAF resistance and MEK inhibitors in some experimental models, other studies suggest that this relationship may be indirect or context-dependent [77,80]. Differences in experimental models, tumor heterogeneity, and variable ABCB5 isoform expression may partially explain these inconsistencies. Furthermore, ABCB5 expression may reflect a broader phenotype-switching program rather than indicating it as an independent driver of resistance. These findings demonstrate the need for standardized models and prospective clinical validation [72].

In addition to isoform heterogeneity, potential ABCB5 subtype-specific expression across melanoma variants remains underexplored. Most studies investigating ABCB5 expression and function have focused on cutaneous melanoma arising on intermittently sun-exposed skin; limited data are available regarding its role in less common subtypes. Given the profound differences in melanoma subtypes’ immune contexture, driver mutations, and therapeutic responses, ABCB5 expression and its functional relevance may be subtype-specific. Currently, robust comparative analyses of ABCB5 expression in acral, mucosal, and CSD melanoma are lacking, revealing an important gap in the literature [33,53,55].

### 8.5. Therapeutic Strategies

Most therapeutic approaches targeting ABCB5 have been explored in preclinical models, and evidence derived specifically from melanoma contexts remains limited. Understanding ABCB5 and its implications for melanoma has opened new therapeutic avenues. Because ABCB5 is an essential regulator of MSCs and plays a role in drug efflux, it is a promising therapeutic target. Inhibiting ABCB5 could increase intracellular drug concentration and reduce cells’ ability to evade both treatment and the immune response. Monoclonal antibodies targeting ABCB5 could be very effective as they could recognize the ABCB5 epitope, enabling the selective removal of ABCB5+ stem cells and, therefore, reducing recurrence and metastatic risk. Preclinical studies of both inhibitors and monoclonal antibodies are ongoing [85,86].

Combined therapy is the most promising direction for new treatments. Chemotherapy, together with ABCB5 inhibitors, could result in maintaining the intracellular concentration of treatment drugs and lead to tumor death. Immunotherapy could also reduce ABCB5+ cells’ immunomodulatory capacity, making tumors more susceptible to immune attack. Combinations of known targeted therapies, such as anti-BRAF medications, could prevent the proliferation of resistant cells and reduce recurrence. Future studies should pursue the development of targeted therapy, explore ABCB5 in liquid biopsies, and integrate immunotherapy with agents that regulate ABCB5 expression, such as miRNA [84,87].

To make the most of these promising avenues, the clinical feasibility of ABCB5 as a predictive or therapeutic biomarker in melanoma requires prospective validation in well-designed clinical studies. Its expression may contribute to patient stratification or treatment selection; any such applications should be approached with caution.

## 9. Conclusions

Unlike previous reviews focusing broadly on melanoma resistance or stem-like tumor cells, this work specifically synthesizes current evidence on ABCB5 within the context of immune modulation, therapy resistance, and tumor plasticity. Current biomarkers provide useful but incomplete information regarding tumor behavior and treatment response. In this context, ABCB5 offers a complementary perspective by reflecting tumor cell plasticity, stem-like phenotypes, and microenvironmental interactions that are not shown by existing markers. Accumulating experimental and correlative clinical evidence suggests that ABCB5 is associated with aggressive disease, therapy resistance, and immune evasion. Translation of ABCB5 into clinical practice requires several steps, including standardization of detection methods, clarification of isoform-specific expression, and validation in large patient cohorts. In the future, ABCB5 should be evaluated in prospective, biomarker-driven clinical studies.

## Figures and Tables

**Table 1:** ABCB5-associated mechanisms and clinical relevance in melanoma.

Mechanism	Effect	Clinical Relevance
Drug efflux	↓ Intracellular drug concentration	Chemoresistance (preclinical evidence)
PI3K/Akt pathway activation	↑ Tumor cell survival, ↓ apoptosis	Promotes tumor progression (preclinical evidence)
BCL-2 modulation	Anti-apoptotic shift	Promotes tumor progression (preclinical evidence)
miR-145 suppression	↑ ABCB5 expression	Resistance in advanced melanoma (preclinical/translational evidence)
Cytokine secretion	Immunosuppressive microenvironment	Poor prognosis, enhanced angiogenesis (clinical association)
PD-L1 upregulation	T-cell inhibition	Immune evasion (preclinical/translational evidence)
Association with aggressive phenotype	↑ Recurrence, ↓ overall survival	Prognostic biomarker (clinical association)
Potential therapeutic target	Monoclonal antibodies, inhibitors	Preclinical studies ongoing

Abbreviations: ↓ indicates decrease/downregulation, ↑ indicates increase/upregulation.

## Data Availability

No new data were created or analyzed in this study. Data sharing is not applicable to this article.

## Abbreviations

The following abbreviations are used in this manuscript:

AJCC	American Joint Committee on Cancer
WHO	World Health Organization
BRAF	B-Raf proto-oncogene, serine/threonine kinase
MSCs	Melanoma stem cells
